# The Epidemiology and Outcome of Biliary Atresia: Saudi Arabian National Study (2000–2018)

**DOI:** 10.3389/fped.2022.921948

**Published:** 2022-07-18

**Authors:** Abdulrahman Al-Hussaini, Mohammed Abanemai, Homoud Alhebbi, Omar Saadah, Razan Bader, Ahmed Al Sarkhy, Maher Alhatlani, Hana Halabi, Ahmed Aladsani, Mohammed AlEdreesi, Sami Wali, Talal Alguofi, Khalid Al-drees, Zahid Arain, Badr Al Saleem, Ali Asery, Sinan Holdar, Sami Alrashidi, Fahad Alsayed, Sulaiman Aldhalan, Amira NasserAllah, Rawabi Alghamdi, Faisal Alhaffaf, Ahmed AlAwfi, Abdulrahman AlSweed, Ali Alshamrani, Manal AlShaikh, Anjum Saeed, Heba Assiri, Muhammed Salman Bashir

**Affiliations:** ^1^Division of Pediatric Gastroenterology, Children's Specialized Hospital, King Fahad Medical City, Riyadh, Saudi Arabia; ^2^College of Medicine, Alfaisal University, Riyadh, Saudi Arabia; ^3^Prince Abdullah Bin Khaled Celiac Disease Research Chair, Department of Pediatrics, Faculty of Medicine, King Saud University, Riyadh, Saudi Arabia; ^4^Department of Pediatrics, King Faisal Specialist Hospital and Research Center, Riyadh, Saudi Arabia; ^5^Division of Pediatric Gastroenterology, Prince Sultan Military Medical City, Riyadh, Saudi Arabia; ^6^Division of Pediatric Gastroenterology, Department of Pediatrics, Faculty of Medicine, King Abdulaziz University, Jeddah, Saudi Arabia; ^7^Multi-Organ Transplant Center, King Fahad Specialist Hospital, Dammam, Saudi Arabia; ^8^King Fahad Specialist Hospital, Dammam, Saudi Arabia; ^9^Gastroenterology Division, Department of Pediatrics, King Saud University Medical City, King Saud University, Riyadh, Saudi Arabia; ^10^Al Imam Abdulrahman Bin Faisal Hospital, Ministry of National Guard Health Affairs, Dammam, Saudi Arabia; ^11^Maternity and Children's Hospital, Makkah, Saudi Arabia; ^12^Department of Pediatrics, College of Medicine, Imam Abdulrahman Bin Faisal University, Dammam, Saudi Arabia; ^13^Specialty Pediatrics Division, Women and Children's Health Institute, Pediatric Gastroenterology, Johns Hopkins Aramco Healthcare, Dhahran, Saudi Arabia; ^14^Organs Transplant Centre, King Faisal Specialist Hospital and Research Center, Riyadh, Saudi Arabia; ^15^Department of Pediatrics, King Abdulaziz Medical City, Ministry of National Guard Health Affairs, Riyadh, Saudi Arabia; ^16^Division of Pediatric Gastroenterology, Department of Pediatrics, Royal Commission Hospital, Jubail, Saudi Arabia; ^17^Division of Pediatric Gastroenterology, King Saud Medical City, Riyadh, Saudi Arabia; ^18^Department of Biostatistics, Research Services Administration, Research Center, King Fahad Medical City, Riyadh, Saudi Arabia

**Keywords:** biliary atresia, neonatal cholestasis, liver transplantation, Arabs, Kasai portoenterostomy

## Abstract

**Background:**

The epidemiology and outcomes of biliary atresia (BA) have been well-documented in national cohorts from two main ethnicities, namely, the Asian Orientals and Caucasians, with incidence ranging from 1 in 5,000 to 1 in 9,000 live births in East Asia and 1 in 15,000 to 19,000 live births in Europe and North America.

**Objective:**

We report the first nationwide BA study outside North America, Europe, and East Asia to describe the epidemiology and outcomes of BA in Saudi Arabia.

**Methods:**

A national database of BA cases diagnosed between 2000 and 2018 was analyzed. We assessed clearance of jaundice (bilirubin <20 μmol/L) in all cases that underwent Kasai portoenterostomy (KPE). We then estimated survival using the Kaplan–Meier method with endpoints of liver transplantation (LT), death, or survival with native liver (SNL).

**Results:**

BA was diagnosed in 204 infants (106 females; 10% pre-term). The incidence of BA was 1 in 44,365, or 2.254 in 100,000 live births (range, 0.5–4 in 100,000). Polysplenia was diagnosed in 22 cases (11%). The median age at referral was 65 days. A total of 146 children (71.5%) underwent KPE at a median age of 70 days. Clearance of jaundice was achieved in 66 of the 146 (45%) infants. The 10-year SNL after KPE was 25.5%, and the overall 10-year estimated survival was 72.5%. The Kaplan–Meier survival curves for patients undergoing KPE at the age of <60, 61–90, and >90 days showed a SNL rate at 51.6, 33, and 12.5%, respectively, at 5 years (*P* < 0.001). The 2-, 5-, and 10-year post-LT survival rates were 92.5, 90.6, and 90%, respectively. Undergoing an initial KPE did not impact negatively on the overall LT survival rate when compared to BA cases that underwent primary LT (*P* = 0.88).

**Conclusion:**

The incidence rate of BA in Saudi Arabia is lower than the incidence reported elsewhere. Late referral of BA cases remains a problem in Saudi Arabia; as a result, the SNL rate was lower than reported by other national registries. Hence, national policies devoted to timely referral and earlier age at KPE are needed.

## Introduction

Biliary atresia (BA) is a rare disorder that results from an inflammatory and fibrosing obstruction of extrahepatic bile ducts and abnormalities of the intrahepatic bile ducts, most probably attributed to multiple factors including genetic, infective, inflammatory, and toxic insults occurring pre- or perinatally (1). Because of the rarity of BA and the limited information from single-center studies, the establishment of national and international registries has been necessary to study epidemiology, management, and outcomes of infants with BA. The epidemiology and outcomes of BA have been well-documented in large national cohorts from East Asia (2, 3), Europe (4–13), and North America (14–16). The incidence of BA varies markedly worldwide ranging from 1 in 5,000 to 1 in 9,000 live births in East Asia (2, 3) and 1 in 15,000 to 19,000 live births in Europe and North America (4–16). Additionally, there was marked variation in the reported outcomes by country based on the referral patterns, timing of Kasai portoenterostomy surgery (KPE), and caseload in every center. The best outcome was reported in Japan and Taiwan, with more than 60% of patients with BA being jaundice-free and surviving with native liver after KPE (17), mainly attributed to the prompt diagnosis and early KPE (before 45 days of age) after implementation of screening for BA using stool color cards (18). The overall survival rate of infants with BA has dramatically improved by improving the results of pediatric liver transplantation (LT), with long-term survival of 80–90%, resulting in BA is the leading indication for LT during childhood, accounting for 40–50% of all the pediatric liver transplants (19).

Published literature on BA from Saudi Arabia is limited to two small-single center studies (20, 21), and data from LT centers show that BA is a major indication for pediatric LT (29–33%) (22, 23). Thus, our initiative to develop a BA nationwide database served to collect large amounts of data and improve the knowledge of epidemiology, natural history, and outcomes of BA. Here, we report the incidence and overall outcomes of BA in Saudi Arabia between 2000 and 2018 and compare our results with other large national studies.

## Patients and Methods

### Study Settings and Design

This is a retrospective, multicenter, nationwide study that included 10 tertiary care governmental hospitals (including 4 LT centers) in different regions across Saudi Arabia. Saudi Arabia consists of 13 provinces; the 10 hospitals are located in the three most populated provinces, namely, Central (five hospitals), Eastern (three hospitals), and Western (two hospitals). In Saudi Arabia, the national health system guarantees free-of-charge services and access to healthcare for all citizens in the 13 provinces. The healthcare system is designed so that tertiary care is highly centralized to these three main provinces that receive referrals of complex cases from the remaining 10 provinces.

### Study Population

We identified all cases of BA referred to each of the 10 hospitals between 2000 and 2018. Inclusion criteria were as follows: (1) having the diagnosis of BA made based on clinical, biochemical, and radiological findings; liver histology; and surgical findings, all consistent with BA, with other causes of neonatal cholestasis ruled out and (2) born and living in Saudi Arabia between 2000 and 2018. The definitive diagnosis was made when cholangiography failed to show a patent biliary tree. In cases where cholangiography was not undertaken, the diagnosis of BA was confirmed by operative findings at subsequent LT and histology of the excised extrahepatic biliary remnant typical of BA. Patients were excluded if (1) the diagnosis of BA was not confirmed or (2) they were diagnosed and had their KPE at another center outside Saudi Arabia. Non-Saudis diagnosed with BA inside Saudi Arabia during the study period were included when analyzing the outcomes but were excluded during incidence analysis. The principal investigator reviewed all the included BA cases to verify the diagnosis of BA, ensure accuracy and completeness, and eliminate duplicated BA cases seen in more than one of the 10 centers.

### Data Collection

Data were collected using a standardized data collection form and entered into an electronic database. The following data were collected retrospectively: date of birth, gender, birth weight, gestational age (prematurity was defined as gestational age <37 weeks), presence of BA splenic malformation syndrome (BASM), date of referral to a pediatric gastroenterologist, date of KPE if performed, time to clear jaundice, whether LT was performed, date of LT, date and status at the last follow-up, and final outcome.

### Study Outcomes

The primary outcomes of the study were as follows: (1) success of KPE (defined as clearance of jaundice and total serum bilirubin <20 mmol/L any time after KPE), (2) survival with native liver (SNL) [ending at death, LT, or the last follow-up], and (3) overall survival (ending at death or the last follow-up with or without LT). All the patients had a minimum of 24 months of follow-up.

### Ethical Considerations

The study has been approved by the local institutional review board log number 16-001 at King Fahad Medical City and the ethics committees in all hospitals.

### Statistical Analysis

Quantitative variables with normal distribution are expressed as mean and standard deviation (SD), quantitative variables with non-normal distribution are expressed as median and range, and absolute number and percentage are used for categorical variables. We determined the incidence of BA per 100,000 live births per year. Survival curves were calculated according to the Kaplan–Meier method, and comparisons were done with a Cox proportional hazards model and log-rank test. Outcome analyses were performed after stratifying patients for age at the time of KPE at ≤60, 61–90, and >90 days.

## Results

Out of 248 BA cases initially submitted by the 10 hospitals, 28 were duplicates and 16 were excluded because of the insufficient information to make a diagnosis of BA. The data of the remaining 204 patients were reviewed, and the outcomes were analyzed.

### Incidence of Biliary Atresia

**Figure 1:**
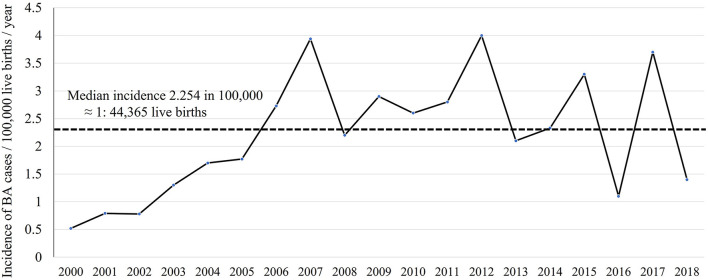
Incidence of biliary atresia cases per 100,000 live births per year.

**Figure 2:**
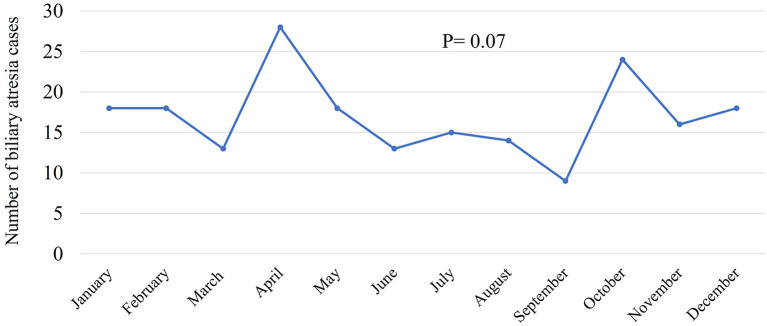
Incidence of biliary atresia cases per month.

Between 2000 and 2018, 7,938,287 Saudi infants were born according to the General Authority for Statistics in Saudi Arabia (24). Of the 204 infants with BA, 179 were Saudi. Therefore, the incidence of BA was 2.254 in 100,000 live births which was approximately equal to 1 in 44,365 live births. The annual number of infants with BA varied between 3 and 17, and the incidence rate ranged from 0.5 to 4 in 100,000 live births. Figure 1 shows the annual incidence of BA during the investigated period. Figure 2 shows the number of BA cases per month, demonstrating a lack of seasonality.

### The General Characteristics of the Study Cohort (*n* = 204)

The female-to-male ratio was 1.08:1. The mean birth weight was 2.83 ± 0.55 kg, and the mean gestational age was 37.6 ± 2.12 weeks. The gestational age was unknown in 47 (23%); of the remaining 157 infants, 118 (75%) were term, 16 (10%) were pre-term, and 22 (14%) were intrauterine growth retarded. The BASM phenotype was diagnosed in 22 patients, representing 11% of the reported cases. No single case of intracranial hemorrhage was observed. The median age at referral to pediatric gastroenterologist was 65 days (range, 7–245 days), and the median age at KPE was 70 days (range, 12–186 days). The median time from the day BA case came to the attention of the pediatric gastroenterologist to the date of KPE was 11 days (range, 1–60 days). All cases were treated initially with ursodeoxycholic acid (20 mg/kg/day, divided twice a day) and supplied with fat-soluble vitamins. After KPE, the use of ursodeoxycholic acid and antibiotics was fairly consistent across the centers but for the variable duration and different regimens. Steroid use was infrequent; only 45 patients (30%) received methylprednisolone or prednisone in different protocols.

### Study Outcomes

**Figure 3:**
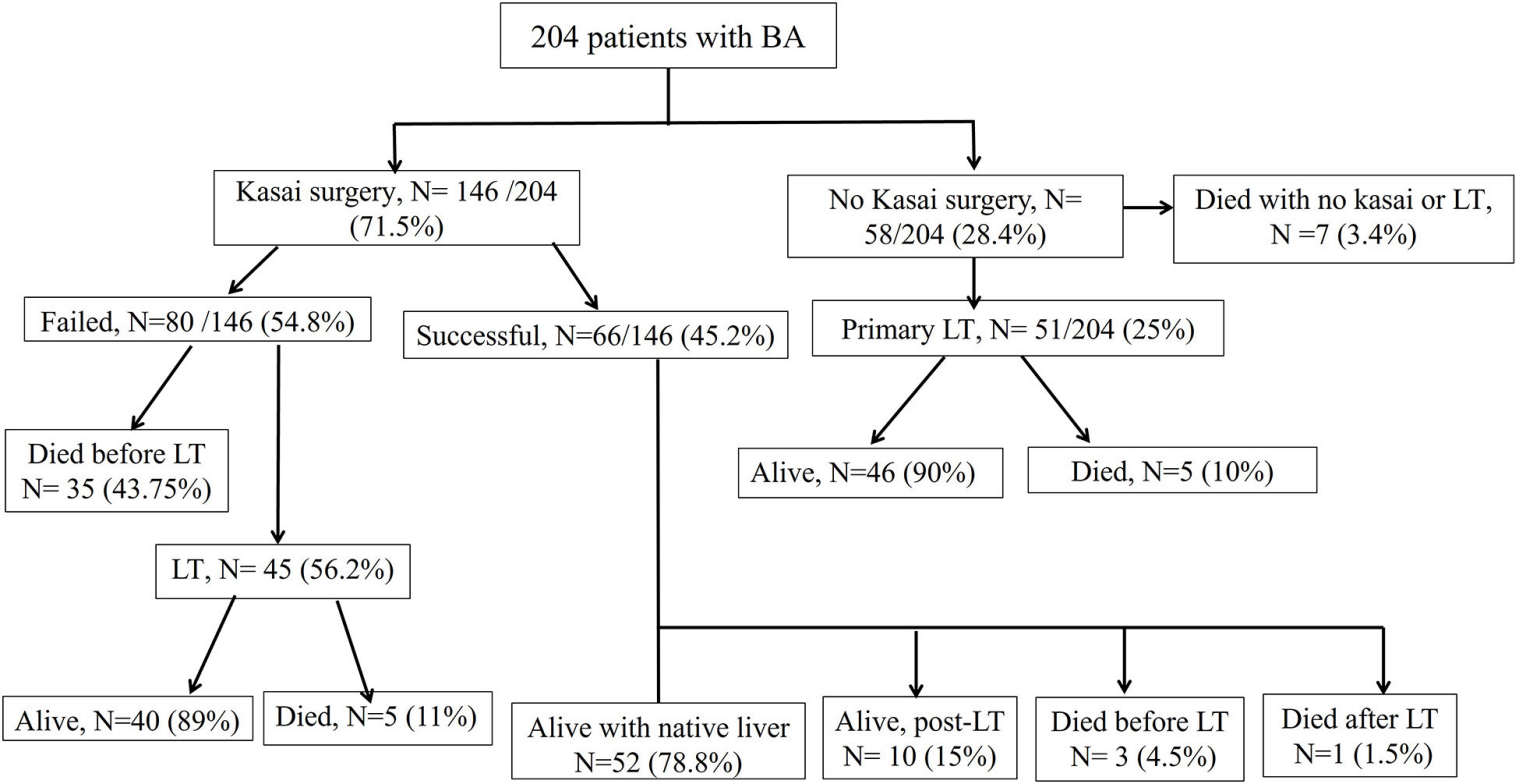
Management and outcomes of the 204 biliary atresia cases.

Figure 3 illustrates the treatment and outcome of the 204 BA patients. The overall average age of the study patients at the last follow-up was 7.2 ± 6 years.

**Figure 4:**
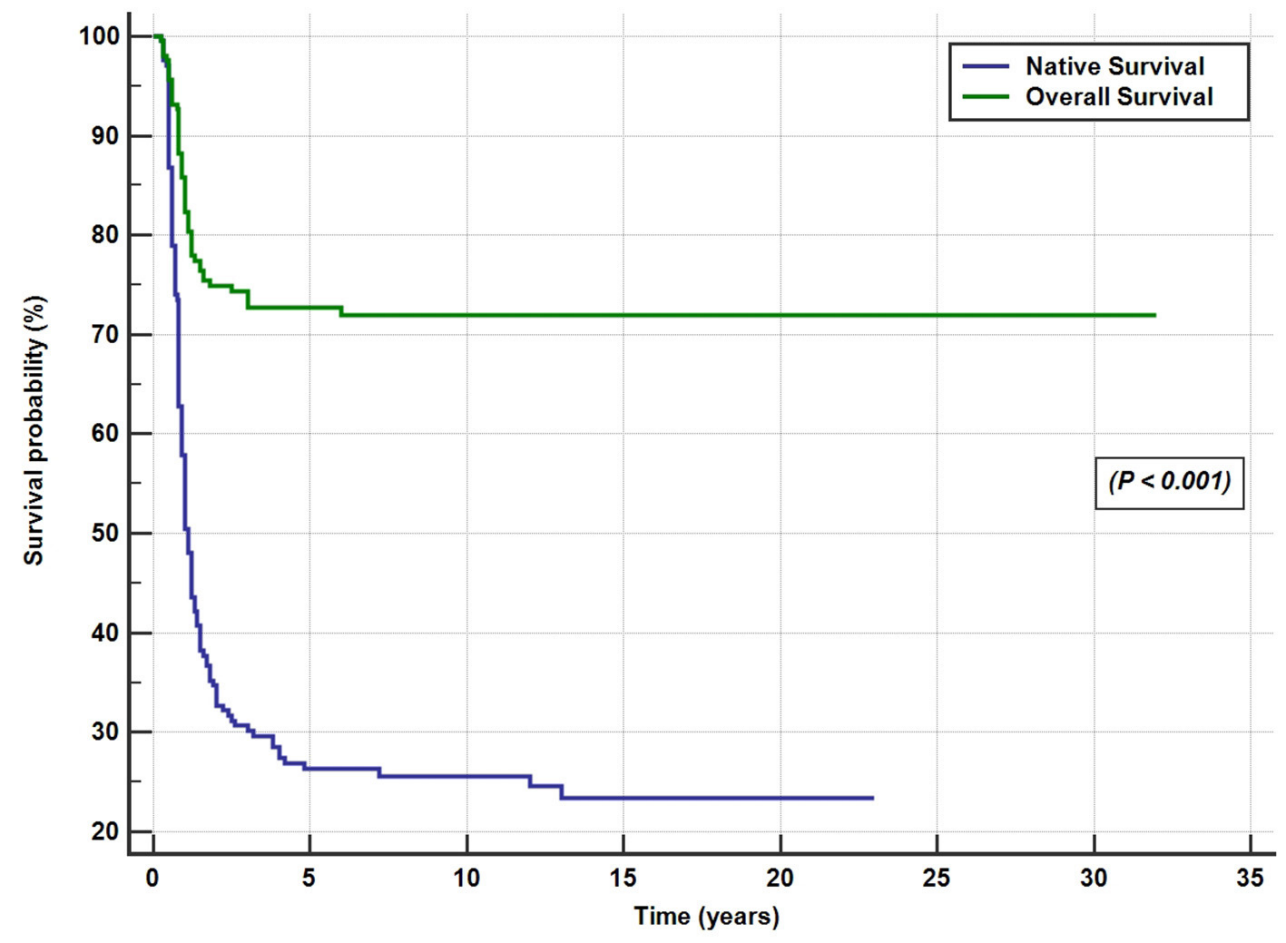
Overall and native liver survival Kaplan–Meier curves for the 204 biliary atresia cases.

**Figure 5:**
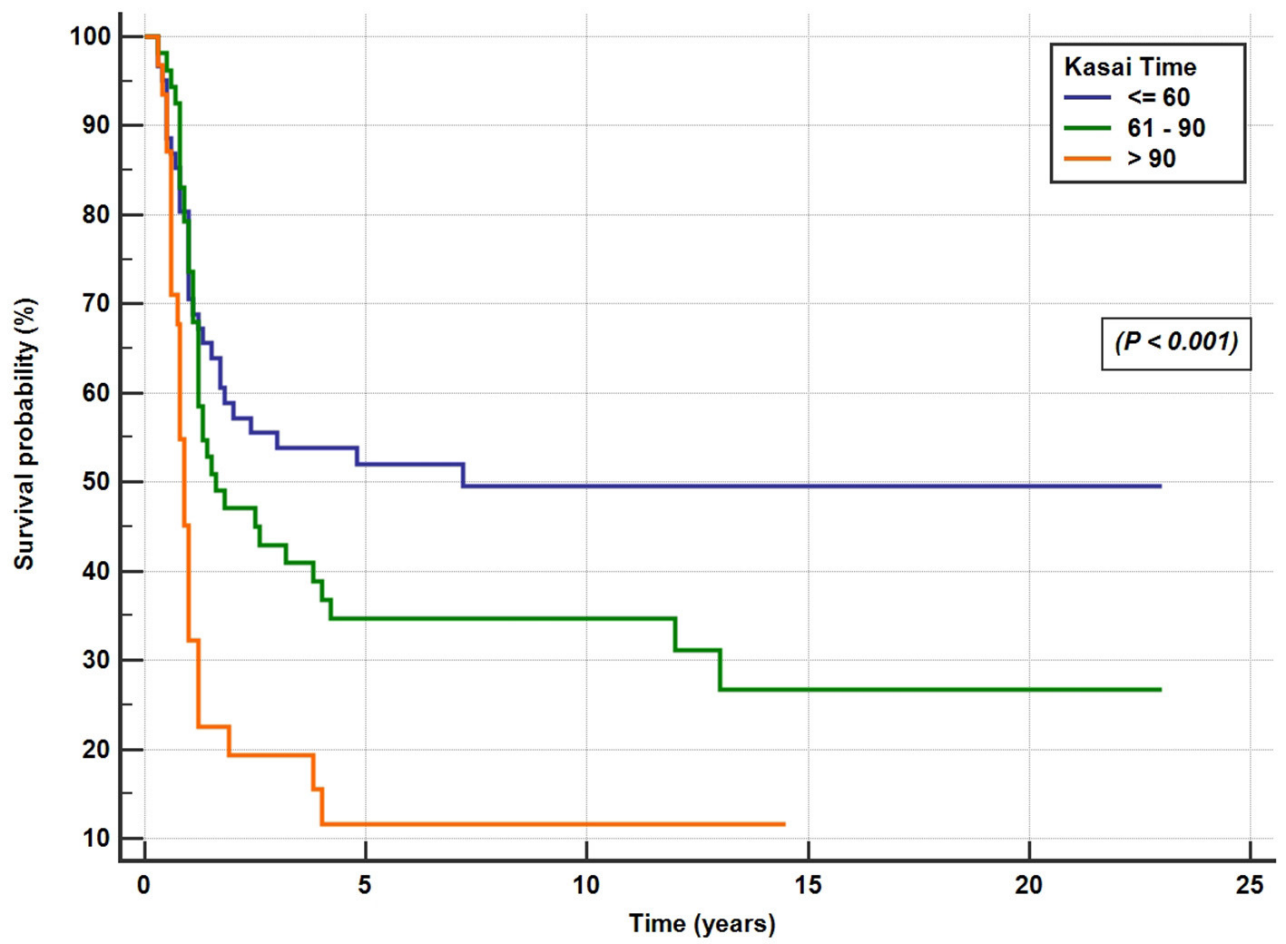
Post-Kasai operation native liver survival rate for the patients who underwent the Kasai surgery at the age of <60 days, 61–90 days, and >90 days.

1. Post-KPE Surgery
  A total of 146 patients (71.5%) underwent KPE at a median age of 70 days (range, 12–186 days). The KPE procedures were performed in all the 10 participating centers; three centers with average caseloads of 1/year (total of 66 cases) and 7 centers with caseloads of <1/year (total of 80 cases). Of the 146 BA patients (42%), 61 underwent KPE before the age of 60 days, 53 (36%) between the ages of 60 and 90 days, and 32 (22%) underwent KPE after the age of 90 days. A total of seven patients (3.5%) presented late with advanced liver disease and died on the waiting list for LT. Of the 51 patients, 31 referred for primary LT presented late (median 120 days) and 21 patients presented before 90 days of age (median 60 days). The median age at referral differed significantly between patients who received KPE (58 days, range 3–180 days) and those who did not (100 days, range 14–245 days; *p* < 0.001). The median duration to the resolution of jaundice in the 66 successful surgeries was 14 weeks (range 2–52 weeks). Of all the 204 patients with BA, SNL was 35, 27, and 25.5% at 2, 5, and 10 years, respectively (Figure 4). Kaplan–Meier survival curve analysis showed that patients undergoing KPE at the age of <60 and 60–90 days survived with their native liver at 2, 5, and 10 years significantly better than patients operated after 90 days (*p* < 0.001; Figure 5).
2. Liver Transplantation
  A total of 107 of the 204 BA patients (52.5%) underwent LT. In 52% (56/107), LT followed KPE (median age 1.2 years, range 0.5–13 years), whereas in 51 cases (48%), LT was performed as a primary surgery (median age 0.8, range 0.5–2.2 years). The average age at the last follow-up of 107 BA patients post-LT was 8.26 ± 5.8 years. At the end of the entire study period, 96 of the 107 BA patients (90%) were alive and 11 died (10%). The 2-, 5-, and 10-year post-LT survival rates were 92.5, 90.6, and 90%, respectively. Performance of the initial Kasai operation did not significantly alter the overall post-LT survival with the 2-, 5-, and 10-year post-KPE transplantation survival rates of 93, 91, and 89.2%, respectively (*p* = 0.88).
3. Overall Survival Rates
  At the end of the study period, 148 of the 204 patients (72.5%) were alive [52 cases (25.5%) with native liver and 96 (47%) post-LT] and 56 BA patients died (27.5%; Figure 4).

## Discussion

This is the first nationwide BA study outside North America, Europe, and Far East Asia to describe the epidemiology and outcome of BA. Our data show some differences and similarities to data from the main BA registries. First, the incidence of BA in Saudi Arabia (1 in 44,000) is lower than the rates reported in Europe and North America (1 in 12,000–19,000 live births) and East Asia (1 in 2,700–1 in 9,400 live births) (25, 26). Second, the median age at referral to pediatric gastroenterologist (65 days) and the age at performance of KPE (70 days) were later than those reported by other national studies worldwide (25, 26). Third, as a result of the delay in referral and performance of KPE, the SNL rates at 2- and 5-year post KPE (35 and 27%, respectively) were lower than those reported by most of the international registries, but the 10-year SNL (25.5%) was similar to data from North America and Europe. On the contrary, the overall survival (72.5%) and post-LT survival rates (90%) at the end of the study period (20 years) compare favorably with the international data, likely reflecting the success of LT programs in Saudi Arabia. Also similar to other large national studies, the age at KPE performance was a significant predictor of outcome.

Our calculated low incidence of BA in Saudi Arabia supports our previous observation that BA contributed to only 5% of 450 Saudi infants with cholestasis (20), as compared to 20–30% in most parts of the world (27). Based on the 1 in 44,000 incidence (2.25 in 100,000 live births) and average annual 417,804 live births, we estimate an average 10 new cases each year, as compared to 18–22 new cases in Canada and 40–45 new cases in the United Kingdom (28). The annual incidence curve of BA in Saudi Arabia (Figure 1) showed a steep rise early in the study period (2000–2005); this could be a true increase in incidence or due to improved diagnosis and referral of BA cases. After 2006, there were three prominent peaks in incidence in 2007, 2012, and 2017 (3.7–4 in 100,000 live births with an estimated 15–17 new cases each year), an observation that might signify environmental factors such as viral infection. We analyzed our data from seasonal aspects but could not identify significant time clustering in line with most of the Western and East Asian studies (29); however, this has been controversial as few studies from the United States showed significant clustering of BA from August to October (29) and December to March (14). Other demographic characteristics in Saudi Arabia, such as the rate of BASM (10.8%) and prematurity (10.2%), were comparable with the findings of previous studies that showed BASM in 8–14% (26) and prematurity in 13% (30). We observed a slight but insignificant female predominance in contrast to most of the Western and East Asian studies that showed significant female predominance (16, 31).

The results of KPE for BA are usually evaluated by looking at the rates of clearance of jaundice and SNL. The clearance of jaundice in our data (45%) compares favorably with several large European studies (34–55%) (23); however, the SNL rate was inferior to the other international registries due to several factors. The SNL is strongly linked to the age at the KPE, which in turn depends on the referral time and the time needed to confirm the diagnosis of BA. In Saudi Arabia, in addition to the delayed referral time, the median time taken to confirm the diagnosis and perform KPE (11 days) is also longer than the 5–7 days of duration recommended to make a prompt BA diagnosis and performance of KPE. Furthermore, it is noteworthy that only 71.5% of the 204 BA patients underwent KPE, in contrast to 89–98% in other large national studies (23), which could be an additional reason for the low SNL rate. Late presentation was the main reason for the referral of 51 cases for primary LT (median 120 days); however, 21 of them presented before 90 days of age (median 60 days). It is possible that some physicians in our country have a low threshold for early LT due to the lower success rate of KPE after 60 days of age and that undergoing an initial KPE could negatively impact the overall LT survival rate (32). In our study, of the 53 patients who underwent KPE between the ages of 60 and 90 days, 33% were alive with the native liver at 5 and 10 years. In addition, our data, supported by others (30, 33–36), clearly show that undergoing an initial KPE did not impact negatively on the overall LT survival rate. Hence, BA patients presenting between the ages of 60 and 90 days should not be denied KPE. However, a more debatable issue is whether KPE is a justified intervention for patients who present after the age of 3 months. Although the 5-year SNL rate of the 28 patients who underwent KPE after 90 days in our study was only 12.5%, similar to the 15% rate in the Canadian study (16), other studies reported a better SNL rate of 25% (37) and 40% (12, 38). Therefore, in infants with acquired type BA presenting after the age of 90 days with well-compensated liver disease, the investigators from these studies supported the sequential management approach with an initial KPE followed by LT if the liver disease becomes advanced (39). Advocates of this approach argue that, since there is no difference in morbidity or mortality rates between BA patients that have a primary or a secondary LT, extending the native liver survival has clear value in relieving the pressure on a limited pool of donor organs, surviving few more years free of immunosuppression-related morbidities, and allowing patients to complete vaccinations and acquire immunity to the common community infections (5, 38).

Data from different international registries correlates KPE performed before 45 days of life with the best outcomes (25, 26). In our study, the impact of age at KPE on BA outcome is very clear (Figure 5). In fact, the short-term SNL (58% at 2 years) and the long-term SNL (50% at 10 years) in patients who underwent KPE at <60 days were similar to, or even better than, centers from Europe and the United States with the most experience, where corresponding rates of 35–60% were reported, but worse than the results from Far East Asia, where > 60% of patients survived free of LT at 2- and 10-year post-KPE (25, 26). These results emphasize the need to diagnose BA early and to promptly operate on these patients.

Another relevant measure of BA outcome is the overall survival that combines the results of KPE and LT, which is 72.5% in Saudi Arabia. It is notable for a large number of BA patients (22%) who died before LT (38 cases post-KPE and 7 before KPE); the majority died during the period from 2000 to 2010. The first pediatric LT center started in November 1998 (22) and was followed by another center in May 2002 (23). For several years after the launch of LT in Saudi Arabia, there was a severe lack of cadaveric grafts due to cultural reasons and the initial reluctance of the local community to accept living donations. These reasons, together with the high demand for LT in a country with a high prevalence of severe familial liver diseases (40), led to high mortality among the BA cases that failed KPE. Public education and awareness campaigns and the legalization of cadaveric and living donation by the Saudi committee of higher religious scholars have significantly relieved the pressure on a limited pool of donor organs; as a result, the mortality without LT significantly decreased over years. Currently, the pediatric LT centers in Saudi Arabia (mostly living-related donors) are high-volume centers with successful results and long-term survival after LT for BA of 90–95% (22, 23).

Besides the retrospective design of the study with its inherent limitations, the lack of consistency across the centers post-operatively in the prescription of prophylaxis antibiotics, ursodeoxycholic acid, and steroids, precluded a reliable analysis of the independent effects of these medications on the outcome. Also, we could not examine outcomes based on the experience of the individual centers because of the relatively limited absolute numbers of KPE procedures in most of the centers.

## Conclusion

The late referral of BA cases to pediatric gastroenterologists remains a major problem in Saudi Arabia; hence, national policies devoted toward timely referral and earlier age at KPE are needed. Efforts are needed to educate general practitioners and pediatricians to obtain a total and direct bilirubin in a baby with prolonged jaundice (beyond 2 weeks of age) and refer cases with cholestasis promptly to a pediatric gastroenterologist. For a geographically large country such as Saudi Arabia with low incidence rate of BA and high live birth rates, studies to evaluate the efficacy and cost-effectiveness of BA screening programs are needed. Several studies reported that BA caseload experience impacts the outcome with centers doing <5 KPE procedures per year (5) or <2 KPE procedures per year (9) having poor results. In Saudi Arabia, with an estimated 10–17 new BA cases each year and a caseload of one KPE annually, there is a need for a centralized policy to ensure that 3–4 tertiary care centers in the three most populated regions perform the KPE to improve the outcome.

## Data Availability Statement

The raw data supporting the conclusions of this article will be made available by the authors, without undue reservation.

## Ethics Statement

The studies involving human participants were reviewed and approved by the local Institutional Review Board (IRB Log Number 16-001) at King Fahad Medical City, and the IRB committees in every hospital Written informed consent to participate in this study was provided by the participants' legal guardian/next of kin.

## Author Contributions

AA-H, MAb, HAl, OS, RB, AAS, MAlh, HH, AAla, and MAlE contributed to the study conception and design and writing the manuscript. SW, TA, KA-d, ZA, BA, and AAs interpreted data and edited the manuscript. SH, SAlr, FA, SAld, AN, RA, FAlh, AAlA, AAlS, AAls, MAlS, and ASa collected data and reviewed the manuscript. MSB performed the statistical analysis. All authors contributed to the article and approved the submitted version.

## Funding

The authors acknowledge the financial support from Prince Abdullah bin Khalid Celiac Disease Research Chair, Department of Pediatrics, Faculty of Medicine, King Saud University.

## Conflict of Interest

The authors declare that the research was conducted in the absence of any commercial or financial relationships that could be construed as a potential conflict of interest.

## Publisher's Note

All claims expressed in this article are solely those of the authors and do not necessarily represent those of their affiliated organizations, or those of the publisher, the editors and the reviewers. Any product that may be evaluated in this article, or claim that may be made by its manufacturer, is not guaranteed or endorsed by the publisher.
